# Child Sleep Linked to Child and Family Functioning in Children with Down Syndrome

**DOI:** 10.3390/brainsci11091170

**Published:** 2021-09-03

**Authors:** Anna J. Esbensen, Emily K. Schworer, Emily K. Hoffman, Susan Wiley

**Affiliations:** 1 Department of Pediatrics, University of Cincinnati College of Medicine, Cincinnati, OH 45267, USA; Susan.Wiley@cchmc.org; 2 Division of Developmental and Behavioral Pediatrics, Cincinnati Children’s Hospital Medical Center, Cincinnati, OH 45229, USA; Emily.Schworer@cchmc.org (E.K.S.); Emily.Hoffman1@cchmc.org (E.K.H.)

**Keywords:** Down syndrome, trisomy 21, sleep, adaptive functioning, family functioning, child

## Abstract

Sleep problems have a bi-directional impact on the daytime performance of children, parental well-being, and overall family functioning in the general population. Children with Down syndrome (DS) are at a high risk of sleep problems, yet the relationship between sleep problems, adaptive functioning, and family stress in children with DS is not well documented. We examined the relationship between sleep (i.e., duration and quality) and child and parent/family functioning. Sixty-six children with DS wore an actigraph for a week to assess their sleep duration and sleep efficiency. Their parents completed ratings on child sleep duration and parasomnias, child adaptive functioning, parental depression and sleep, and family stress. The parents’ reports of their children’s sleep duration were associated with parental depressive symptoms. The parents’ reports of their children’s restless sleep behaviors were associated with poorer performances in child-compliant/calm behaviors, worse parental sleep, and negative parental feelings and sibling relationships. The findings from actigraph measures of the children’s sleep demonstrated that greater sleep efficiency was associated with greater child adaptive functioning and fewer parental depressive symptoms. The study findings provide preliminary evidence that sleep problems are related to child adaptive functioning, parental functioning, and family stress in children with DS.

## 1. Introduction

Individuals with Down syndrome (DS) often have a comorbid intellectual disability, a unique genotypic and phenotypic profile associated with their extra chromosome 21, and an increased risk of various medical and behavioral comorbidities. Coexisting medical conditions that are common in individuals with DS include congenital heart defects, sleep disturbances (including sleep apnea), hypothyroidism, hearing loss, and Alzheimer’s disease [1,2]. These medical conditions are commonly associated with negative outcomes for children with DS, including increased behavior concerns, worse executive functioning outcomes, and an increased risk of mental health symptoms [3,4,5,6,7]. The families of children with DS are also susceptible to greater parental and family stress, and connections have been made between family functioning and child characteristics in DS [3,8,9,10]. Because of these increased risks for children and families of children with DS, there is a need to investigate the relation between specific medical conditions (i.e., sleep disturbances) and overall child and family functioning in order to improve supports for the family system in this population.

Sleep disturbances, including difficulties with sleep onset and maintenance, and premature awakening, are reported in 56–69% of children with DS [11,12,13]. In the general population, sleep disturbances are related to negative outcomes in children, specifically behavioral regulation and neurobehavioral functioning [14,15,16,17,18]. These findings are similarly present among children with intellectual disabilities [19,20,21,22,23,24,25]. There is a growing body of research in the field of DS supporting a relationship between shorter sleep periods or greater sleep disturbances and negative outcomes for behavior and executive functioning, both at home and at school [7,26,27].

Sleep problems are also associated with negative outcomes in child adaptive behaviors, including daily living skills. In children with typical development and children with autism spectrum disorder, sleep behaviors and sleep-disordered breathing are associated with poorer daytime behavior and adaptive skills [28,29,30,31]. Further, these findings have been found to have worse outcomes in adaptive functioning for children with DS and sleep concerns in comparison with their typically developing peers [32]. Specifically, sleep concerns contributed to worse outcomes for personal care, interpersonal relationships, school performance, fitness, and communication, beyond those contributed by the presence of DS. Additionally, in a sample of children with DS with a median age of 9 years, those with moderate-to-severe obstructive sleep apnea had lower conceptual adaptive behavior scores as compared to children with DS without obstructive sleep apnea [33], providing further evidence of a potential relationship between sleep disturbance and daytime adaptive behaviors in DS. Thus, there is a need to understand the full complexity of how adaptive functioning outcomes are associated with sleep problems in children with DS.

Beyond the negative impact of sleep disturbances on children’s adaptive functioning, there is also the negative impact of sleep problems on the parental well-being and family functioning reported in the general population [34,35,36,37]. There is some preliminary evidence to suggest the downstream impact of sleep problems on parents and family from children with an intellectual disability. Greater parental stress, poorer parental well-being, and poor parenting behaviors (high discipline, low affection) among parents of children with intellectual disability have been shown to be associated with child sleep problems [22,23,38]. Among these parents, their child’s poor sleep had a negative impact on their own [22,23,38]. There is preliminary evidence to support the association between child sleep disturbances and parental well-being in families of children with DS [39]. To better design supports and interventions, there is a need to understand the relationship between child sleep functioning and parental and family functioning within the field of DS.

To address this gap, the present study was designed to examine the relationship between child sleep problems and child or parent/family functioning among families of children with DS. Child sleep problems were assessed using both subjective (parental report) and objective (actigraphy) measures of sleep duration and sleep quality. Parents also reported on the functioning of both the child and the parent and family. We generated three hypotheses to investigate the relation between child sleep and child adaptive functioning, parental functioning, and family functioning. First, we hypothesized that parental report and actigraph-measures of child sleep duration and sleep quality would be related to lower *child adaptive functioning* on parental report measures. Second, we hypothesized that parental report and actigraph-measures of child sleep duration and sleep quality would be related to poorer *parental functioning*, as measured by depressive symptoms and sleep behaviors. Third, we hypothesized that parental report and actigraph-measures of child sleep duration and sleep quality would be related to poorer *family functioning*, as measured by indices of parental feelings toward parenting and the impact on siblings.

## 2. Materials and Methods

### 2.1. Participants

As part of the baseline assessments of two larger community-based intervention studies on behavior and sleep, rating forms were completed by the parents of 66 children with DS. Children with DS were between 6 and 17 years of age (*M* = 11.4 years, *SD* = 3.4) and just over half were male (59.1%). Children were primarily Caucasian (84.8%), with 12.1% identifying as African-American and 3.0% as another race. Only 2.0% identified as Hispanic. Over half (53.0%) of the children had a confirmed current diagnosis of obstructive sleep apnea in their medical records. Parents serving as respondents were primarily mothers (93.5%).

### 2.2. Procedures

The parents of children with DS were recruited from medical settings (i.e., a pediatric medical center and a DS specialty clinic) and through newsletters distributed by local DS associations for two community-based intervention studies. The eligibility criteria for the broader studies included having a child with DS between the ages of 6 and 17 years and speaking English as a primary language. The children had a nonverbal mental age of at least 36 months and were required to have at least one behavioral disturbance, both based on their parental report. The exclusion criteria included a clinical diagnosis of autism spectrum disorder, and limited language abilities, motor impairments, or sensory impairments (deafness or blindness) that would preclude the valid administration of the study measures or the neuropsychological assessment in the larger intervention studies.

The children were administered a neuropsychological assessment of cognitive skills. The parents completed forms collecting demographic and medical information, as well as a series of caregiver report forms evaluating child sleep and adaptive behavior, parent sleep and depressive symptoms, and family functioning. All study activities were approved and overseen by the Institutional Review Board at the medical center.

### 2.3. Measures

#### 2.3.1. Child Cognition

The children’s cognitive ability was measured using the Kaufman Brief Intelligence Test, Second Edition (KBIT-2), which is appropriate for individuals aged 4–90 years and recommended for studies of cognition among individuals with DS [40,41,42]. Standard scores on this test have a mean of 100 and a standard deviation of 15.

#### 2.3.2. Child Sleep

The children’s sleep was assessed using both actigraphy and the parental reports. The Micro-Mini Motionlogger Actigraph (Ambulatory Monitoring, Inc.) was worn by children on their non-dominant wrist to objectively infer sleep from their movement patterns. The children wore the actigraph for seven nights, placing the actigraph 30 min before bedtime until 30 min after rising in the morning. A validated sleep-scoring algorithm was used to obtain measures of (1) Sleep Duration: sleep period, the time from when the child first fell asleep to when the child woke up, ignoring waking times within that period; and (2) Sleep Quality: sleep efficiency, defined as the percentage of the sleep period that the child spent in sleep [43,44]. These measures were selected for consistency with prior literature and convergent validity with polysomnography [7,27,45].

The parental reports on the children’s sleep were collected using the Children’s Sleep Habits Questionnaire (CSHQ) [46]. The CSHQ is a 33-item sleep screening instrument that assesses major childhood medical and behavioral sleep disorders. Items are rated on a 3-point ordinal scale from (1) rarely (0–1 time/week) to (3) usually (5–7 times/week). The CSHQ demonstrates strong psychometric properties and convergence in the identification of behavioral sleep problems in children with DS and has demonstrated validity in other pediatric populations that are characterized by intellectual and developmental disabilities [47,48,49,50]. Two subscales assessing sleep problems were used in the current analyses, specifically the subscales of Sleep Duration and Parasomnias, to mirror the objective actigraph measures of sleep duration and sleep efficiency, and for consistency with prior research [7,27].

#### 2.3.3. Child Adaptive Behavior

Adaptive behavior was measured using the Scales of Independent Behavior-Revised (SIB-R) and the Nisonger Child Behavior Rating Form (NCBRF). Both measures are appropriate for use with children with intellectual and developmental disabilities [40,41].

The SIB-R rates children’s adaptive daily living skills and yields a standard score in four domains (motor skills, social interaction/communication skills, personal living skills, and community living skills) and an overall Broad Independence score [51]. Standard scores have a mean of 100 and a standard deviation of 15.

The NCBRF measures adaptive and maladaptive behaviors among children with intellectual and developmental disabilities [52]. Only the two adaptive subscales of the NCBRF, Compliant/Calm (6 items) and Adaptive/Social (4 items), were evaluated in this study. The items are rated on a 4-point scale from Not True (scored 0) to Completely or Always True (scored 3). The item-mean scores are presented to support the interpretation of subscales with a different number of items; thus the range of scores is 0–3.

#### 2.3.4. Parental and Family Functioning

Parental functioning was evaluated using the Center of Epidemiological Studies-Depression Scale Revised (CESD-R) and Pittsburgh Sleep Quality Index (PSQI). The CESD-R is a reliable and valid measure of depression, and is commonly used to assess the well-being of parents of individuals with ID [53,54]. Two subscales were selected for evaluation, specifically the Sleep (3 items) and Think (2 items) subscales. Items are rated on a 3-point scale from Not at All (scored 0) to Nearly Every Day (scored 3) for 2 weeks. Summed scores are presented, thus the range of scores is 0–9 for Sleep and 0–6 for Think.

Items on the PSQI are rated on a 4-point scale from Not During the PastMonth (scored 0) to Three or More Times a Week (scored 3), in addition to providing estimates of the parent’s total time in bed and their total time asleep [55]. The Sleep Duration and Sleep Efficiency subscales were selected for evaluation to mirror the objective and subjective measures of child sleep. Sleep Duration is coded on a 0 to 3 scale dependent on the total amount of sleep obtained, with lower scores representing better sleep (0 = 7 or more hours) and higher scores representing less sleep (3 = less than 5 h of sleep). Sleep Efficiency is coded on a 0 to 3 scale dependent on the ratio of the total time in bed to the total amount of time asleep, with lower scores representing better sleep efficiency (0 = 85% or more) and higher scores representing worse sleep efficiency (3 = less than 65%).

Family stress was measured with the Family Impact Questionnaire (FIQ). The FIQ is designed to measure parental stress beyond the stress of raising a child with a developmental disability, specifically how the child positively and negatively impacts on parenting, social relationships, finances, and, as applicable, siblings, and marriage [56]. The internal consistency of the FIQ is excellent [57]. Items are rated on a 4-point scale from Not at All (scored 1) to Very Much (scored 4), with two items scored on a 1 to 6 scale. The subscales of Negative Feelings toward Parenting (9 items) and Positive Feelings toward Parenting (seven items) were evaluated to assess parental functioning, and the subscale of Impact on Siblings (nine items) was evaluated in order to further assess family functioning. Higher scores reflect a greater endorsement of the subscale, with possible ranges of scores of 9–36 for Negative Feelings and Impact on Siblings, and 7–30 for Positive Feelings.

### 2.4. Data Analysis

The descriptive information and correlations among measures were calculated for the sleep measures and the parental report measures of child and parent/family functioning. Simultaneous or hierarchical linear regressions tested whether actigraphy and parental report measures of sleep duration and quality predicted parental reports of child adaptive functioning. Separate regressions were run for the five subscales of the SIB-R and the two subscales of the NCBRF. Because SIB-R takes into account age and gender, hierarchical methods were not needed. Additionally, hierarchical linear regression tested whether the actigraphy and parental report measures of sleep duration and quality predicted parental reports of parent/family adaptive functioning. Separate regressions were run for the subscales of the CESD-R, two subscales of the PSQI, and three subscales of the FIQ. As age and gender are considered in creation of SIB-R standard scores, age and gender were entered as covariates in the first step in all other hierarchical analyses. In the second step, CSHQ duration and parasomnias, and actigraphy-based sleep period and sleep efficiency, were entered as predictors.

## 3. Results

The descriptive data for the children’s age and cognitive ability, the measures of the children’s sleep, and child and parent/family functioning are presented in Table 1. The correlations between the measures of child sleep and child and parent/family functioning are presented in Table 2. Multi-collinearity was found to not be a concern in regression analyses (tolerance < 0.1, variance inflation factor < 5).

### 3.1. Sleep Predicting Child Functioning

Our first research aim was to address how child sleep relates to parental reports of child adaptive functioning on the SIB-R and NCBRF (see Table 3). Age and gender are considered in the calculation of SIB-R, and thus were not controlled for in these hierarchical analyses. Age and gender were controlled for in predicting the NCBRF subscales, yet neither was significantly related to the child functioning measures in Step 1.

Better actigraph-measured sleep efficiency was related to higher parent ratings on the SIB-R of the children’s Broad Independence (*β* = 0.39, *p* < 0.01), Motor Skills (*β* = 0.35, *p* < 0.01), and Personal Living Skills (*β* = 0.33, *p* < 0.05). The predictor variables accounted for 17–22% of the variance in the child functioning outcomes. Other measures of child sleep did not significantly predict parent ratings on the SIB-R.

The rating of fewer concerns on the CSHQ parasomnia subscale was related to better parent ratings on the NCBRF Compliant/Calm (*β* = −0.39, *p* < 0.05). In addition, better altigraph-measured sleep efficiency was related to better parental ratings on the NCBRF Compliant/Calm (*β* = 0.47, *p* < 0.01) and Adaptive/Social (*β* = 0.57, *p* < 0.01) subscales. In Step 2, age was also a significant predictor of NCBRF Adaptive/Social (*β* = 0.38, *p* < 0.05), with older children reported to have better adaptive/social skills. The predictor variables accounted for 44–50% of the variance in the child functioning outcomes.

### 3.2. Sleep Predicting Parent/Family Functioning

Our second research aim was to address how children’s sleep relates to parent/family functioning on the CESD-R, PSQI, and FIQ (see Table 4). In Step 1, age and gender were controlled for in hierarchical analyses. Age was a significant predictor of Negative Feelings toward Parenting on the FIQ (*β* = −0.31, *p* < 0.05), with parents reporting more negative feelings for younger children. This significant relationship with age was present again in Step 2 (*β* = −0.30, *p* < 0.05). Neither age nor gender was significantly related to any of the other analyzed parental report measures of parent/family functioning.

Shorter sleep duration on the CSHQ (*β* = −0.27, *p* < 0.05) and worse sleep efficiency on actigraphy (*β* = −0.52, *p* < 0.05) were related to more concerns on parental reports of sleep problems on the CESD-R. The predictor variables accounted for 40% of the variance in CESD-R Sleep. The rating of more concerns on the CSHQ parasomnia subscale was related to more concerns reported for sleep efficiency on the PSQI (*β* = 0.36, *p* < 0.05), accounting for 22% of the variance.

Concerns on the CSHQ parasomnia subscale were also related to more Negative Feelings toward Parenting (*β* = 0.25, *p* < 0.05) and Impact on Siblings (*β* = 0.29, *p* < 0.05) on the FIQ, accounting for 17–25% of the variance in family functioning.

## 4. Discussion

This study investigated the relation between parent-rated and actigraph-measured reports of sleep, and child and parent/family functioning in DS. Parental ratings of their childrens’ restless sleep behaviors and actigraph measurements of sleep efficiency were associated with child functioning. Sleep efficiency was found to be the most salient predictor of the children’s adaptive behaviors. Additionally, actigraph-measured sleep efficiency and parental ratings of children’s sleep duration were related to parents’ depressive symptoms. Child parasomnia was also a significant unique contributor to parents’ sleep efficiency, negative feelings towards parenting, and impact on siblings. Taken together, these results corroborate and add to the growing understanding of the broad impact of sleep problems in DS on child, parent, and family functioning.

One notable finding from this study was the association between sleep quality and multiple components of child functioning. Specifically, actigraph-measured child sleep efficiency was associated with independent functioning, motor skills, communication, and personal living. Parental ratings of the children’s restless sleep behaviors were also significantly related to child compliant/calm behaviors. The direction of these relations was in line with our hypothesis that lower sleep quality would be associated with lower child adaptive functioning on parental report measures. These findings corroborate previous observations of the relation between child sleep and broad independent, communication, and personal care skills [32,33], and also extends these findings in connection to motor skills. These findings potentially reflect sluggish movements or difficulties with coordination in the presence of poor sleep quality. In the model predicting adaptive functioning, actigraph-measured sleep efficiency emerged as a predictor of multiple adaptive domains, suggesting that sleep quality may have a substantial impact on the daytime functioning of children with DS. Although this interpretation corresponds with the theoretical design of the study because data were cross-sectional, it is also possible that adaptive behaviors impact sleep efficiency or that there might be bidirectionality in the relation between these two domains. Regardless, recognizing the broader connection between child sleep and adaptive functioning has implications for intervention targets and treatment planning for DS. Sleep education and intervention should be considered when targeting adaptive functioning outcomes in DS. Understanding the full complexity of the relation between adaptive functioning and sleep problems in children with DS should continue to be studied in this at-risk population in order to inform intervention techniques and optimize adaptive outcomes for children with DS.

Children’s sleep was also significantly associated with parents’ depressive symptoms and parental sleep. The parental reports of children’s restless sleep (parasomnia) was negatively associated with parental ratings of their own sleep efficiency, indicating that challenges with children’s sleep likely interrupt parents’ sleep. Specific connections were also identified between parent-rated child sleep duration and both child actigraph sleep efficiency and sleep-related parental depression symptoms. Although child sleep problems likely have a negative impact on parental well-being, it is also possible that the relation is bidirectional, in that parents with more depressive symptoms and poor sleep influence the broader home environment, which in turn affects child sleep. These study findings substantiate the preliminary findings of a relation between child sleep disturbances and parental well-being in DS [39] and the negative impact on parental sleep among parents of children with intellectual disabilities [22,23,38]. Taken together, these results corroborate the findings that children’s sleep duration and quality are related to parental functioning, and that child sleep should be pursued for treatments designed to support parents of children with DS.

In addition to parental functioning, sleep problems were also associated with family functioning. We hypothesized that parent-reported and actigraph measures of children’s sleep duration and quality would be related to poorer family functioning, as measured by indices of parental feelings toward parenting and the impact on siblings. Both negative feelings toward parenting and the impact on siblings were associated with parent ratings of parasomnias, such that more restless sleep behaviors were related to poorer family functioning. This broader impact of sleep on family stress underlines an increased need to attend to sleep treatments, especially given the high rate of concern over sleep problems in DS. Regression models verified this significant association and identified chronological age as a significant predictor of negative feelings towards parenting, with parents of younger children reporting more negative feelings. Although the data are cross-sectional, we can speculate that parents may adapt more easily to challenges that lead to negative feelings in the case of older children, or that older children present fewer challenges that lead to negative feelings. Overall stress may also be lower in parents of older children because of these children’s lower reliance on their parents to help them to sleep as they transition into adolescence. These findings are in line with the associations between sleep and family functioning reported in the general population [34,35,36,37] and extend this information as it applies to DS. Similarly to the child and parent functioning variables, there is also potential for bidirectionality in the association between sleep and family functioning, as suggested by other studies of the impact of family stress on sleep in typically developing adolescents [58]. The implications of children’s sleep for the family system are essential to identify in order to establish treatment plans that include and support families of children with DS.

### Limitations and Future Directions

This study has several strengths, including the multiple methods of sleep measurement used, its corroboration of previous findings in children with DS, intellectual disability, and typical development, and its identification of the connection between a medical concern and an area of challenge in DS. Despite these strengths, this study has several limitations. First, the parent and family measures were all parental report measures and no behavioral observations were conducted to support these results. There was also no measurement of medically assessed obstructive sleep apnea, and medically focused studies are warranted for future research. Furthermore, other medical conditions common in DS (e.g., hypothyroidism, congenital heart defects) should be investigated with regards to child and parent/family functioning to further understand the family impact of specific medical conditions [3]. Finally, it is likely that adaptive behavior also affects parent and family functioning, and while the focus here was on sleep, future work should examine the potential for sleep as a mediator in the connection between adaptive behavior and parent and family outcomes. Despite these limitations, this study helps us better understand how sleep affects child and family characteristics in DS.

## 5. Conclusions

This study provides evidence of the broad impact of children’s sleep on child and parent/family functioning. The results showed a connection between sleep problems, an area of medical concern in DS, and child adaptive functioning, a specific area of challenge for individuals with DS. Associations were also identified between children’s sleep and the components of both parent and family functioning. The findings suggest that children’s sleep problems have implications not only for children’s daily functioning but also the functioning of the family system. Sleep education and training should be strongly considered for future interventions aimed to support children with DS and their families.

## Figures and Tables

**Table 1:** Participant demographics and descriptive statistics for child functioning, parent/family functioning, and child sleep.

	M (SD)	Range of Scores
*Demographics*		
Age	11.4 (3.4)	6–17
KBIT-2 IQ standard score	44.6 (6.2)	40–71
*Child Functioning*		
SIB-R Broad Independent	47.4 (19.5)	3–82
SIB-R Motor	55.1 (19.5)	14–100
SIB-R Social/Communication	59.3 (21.3)	9–102
SIB-R Personal Living	59.2 (20.8)	6–102
SIB-R Community Living	45.0 (19.4)	4–82
NCBRF Compliant/Calm	1.7 (0.5)	0.6–2.8
NCBRF Adaptive/Social	1.6 (0.5)	0.7–2.5
*Parent/Family Functioning*		
CESD-R Sleep	2.8 (2.4)	0–10
CESD-R Think	1.7 (1.7)	0–6
PSQI Sleep Duration	0.8 (1.0)	0–3
PSQI Sleep Efficiency	0.6 (0.9)	0–3
FIQ Negative Feelings toward Parenting	19.7 (5.8)	10–31
FIQ Positive Feelings toward Parenting	21.8 (5.3)	11–31
FIQ Impact on Siblings	15.2 (4.8)	0–25
*Child Sleep*		
CSHQ Sleep Duration	5.2 (1.7)	3–8
CSHQ Parasomnias	9.1 (1.8)	6–14
Actigraphy sleep period (mins)	543.9 (51.9)	422.9–634.2
Actigraphy sleep efficiency (%)	88.3 (7.8)	56.0–98.2

Note: KBIT-2 = Kaufman Brief Intelligence Test, Second Edition; SIB-R = Scales of Independent Behavior-Revised; NCBRF = Nisonger Child Behavior Rating Form; CESD-R = Center of Epidemiological Studies Depression Scale–Revised; PSQI = Pittsburgh Sleep Quality Index; FIQ = Family Impact Questionnaire; CSHQ = Children’s Sleep Habits Questionnaire.

**Table 2:** Correlations between demographic or independent variables and dependent variables.

	CSHQ Sleep Duration	CSHQ Parasomnias	Actigraphy Sleep Period	Actigraphy Sleep Efficiency
Age	0.09	−0.20	−0.15	−0.21
KBIT−2 IQ standard score	0.15	0.08	0.11	0.08
SIB-R Broad Independent	−0.07	−0.10	0.26 **	0.42 **
SIB-R Motor	−0.05	−0.24	0.38 **	0.40 **
SIB-R Social/Communication	0.02	−0.14	0.34 **	0.19
SIB-R Personal Living	−0.14	−0.17	0.29 *	0.35 **
SIB-R Community Living	−0.11	0.03	0.24	0.15
NCBRF Compliant/Calm	−0.03	−0.52 **	0.11	0.29
NCBRF Adaptive/Social	−0.12	−0.48 **	0.24	0.36
CESD-R Sleep	−0.16	0.16	−0.58 ***	−0.51 ***
CESD-R Think	0.20	0.09	−0.01	−0.13
PSQI Sleep Duration	0.20	0.36 *	−0.37 *	−0.22
PSQI Sleep Efficiency	0.23	0.35 *	−0.15	−0.13
FIQ Negative Feelings toward Parenting	0.19	0.29 *	0.06	0.16
FIQ Positive Feelings toward Parenting	−0.07	−0.09	−0.02	−0.03
FIQ Impact on Siblings	0.22	0.25 *	0.10	0.15

Note: * *p* < 0.05, ** *p* < 0.01, *** *p* < 0.001.

**Table 3:** Predicting child functioning from child sleep.

	SIB-R					NCBRF	
	Broad Independent β	Motor β	Social/Communication β	Personal Living β	Community Living β	Compliant/Calm β	Adaptive/Social β
Step 1							
Age	-	-	-	-	-	0.239	0.343
Gender	-	-	-	-	-	0.197	0.132
*R* ^2^ *change step 1*	-	-	-	-	-	*0.090*	*0.129*
Step 2							
Age	-	-	-	-	-	0.210	0.381 *
Gender	-	-	-	-	-	0.149	0.106
CSHQ Duration	−0.022	0.008	0.081	−0.079	−0.125	−0.019	−0.120
CSHQ Parasomnia	−0.116	−0.234	−0.144	−0.208	−0.025	−0.389 *	−0.292
Sleep period	0.177	0.131	0.229	−0.012	0.104	−0.303	−0.213
Sleep efficiency	0.394 **	0.346 **	0.169	0.330 *	0.132	0.468 *	0.565 **
*R* ^2^ *change step 2*	-	-	-	-	-	0.354 *	0.376 *
*R* ^2^ *total*	0.221 *	0.224 *	*0.107*	0.179 *	*0.052*	0.445	0.505

* *p* < 0.05, ** *p* < 0.01; Note: SIB-R *n* = 49–56; NCBRF *n* = 29.

**Table 4:** Predicting family functioning from child sleep.

	CESD-R		PSQI		FIQ		
	Sleep β	Think β	Sleep Duration β	Sleep Efficiency β	Negative Feelings toward Parenting β	Positive Feelings toward Parenting β	Impact on Siblings β
Step 1							
Age	0.259	−0.099	0.050	0.115	−0.312 *	0.228	−0.048
Gender	0.056	−0.046	0.034	0.190	0.076	0.187	0.116
*R* ^2^ *change step 1*	*0.071*	*0.012*	*0.004*	*0.050*	0.107*	*0.081*	*0.016*
Step 2							
Age	0.206	−0.147	0.017	0.139	−0.298 *	0.237	0.010
Gender	0.085	−0.038	0.100	0.262	0.112	0.184	0.138
CSHQ Duration	−0.269 *	0.172	0.115	0.173	0.217	−0.093	0.225
CSHQ Parasomnia	0.081	−0.085	0.249	0.356 *	0.254 *	−0.031	0.287 *
Sleep period	−0.176	0.066	−0.195	0.037	−0.127	0.001	0.043
Sleep efficiency	−0.523 ***	−0.130	−0.209	−0.065	0.134	−0.010	0.190
*R* ^2^ *change step 2*	0.328 **	*0.057*	*0.183*	*0.175*	0.148 *	*0.010*	*0.158*
*R* ^2^ *total*	0.399	0.069	0.187	0.225	0.255	0.091	0.174

* *p* < 0.05, ** *p* < 0.01, *** *p* < 0.001; Note: CESD-R *n* = 44; PSQI *n* = 44; FIQ *n* = 57–59.

## Data Availability

The data presented in this study are available on request from the corresponding author. The data are not publicly available due to privacy restrictions.
